# Effect of HNO_3_ and H_2_SO_4_ on the Paddy Ecosystem: A Mesocosm Study with Exposure at PNEC and HC_50_ Levels

**DOI:** 10.3390/ijerph17145244

**Published:** 2020-07-21

**Authors:** Minseok Park, Wonjae Hwang, Jino Son, June Wee, Kijong Cho, Seunghun Hyun

**Affiliations:** Department of Environmental Science and Ecological Engineering, Korea University, Seoul 02841, Korea; asithinkyou@korea.ac.kr (M.P.); hwj0145@korea.ac.kr (W.H.); rogix2001@korea.ac.kr (J.S.); dnlwns@korea.ac.kr (J.W.); kjcho@korea.ac.kr (K.C.)

**Keywords:** rice (*Oryza sativa*), paddy mesocosm, acid spill, nitric acid, sulfuric acid

## Abstract

Paddy mesocosms comprising of rice (*Oryza sativa*), snail (*Pomacea canaliculata*), and worm (*Tubifex tubifex*) were used to assess the damage caused by two acids (HNO_3_ and H_2_SO_4_) at predicted no-effect concentration (PNEC) and hazardous concentration for 50% of species (HC_50_) levels. In the fourth week, the fresh weight and shoot height of *O. sativa* at H_2_SO_4_-HC_50_ were reduced by 83.2% and 30.3%, respectively. Wilted leaves (%) at HC_50_ were approximately twice that at PNEC. No *P. canaliculata* and *T. tubifex* were recovered at HC_50_. At H_2_SO_4_-PNEC, the length and weight of *P. canaliculata* were reduced by 7.4% and 25.9%, respectively, whereas fewer adult (46.5%) and juvenile (84%) *T. tubifex* were recovered. In the 20th week, rice growth and productivity were correlated with initial pH (pH_i_) and nitrogen levels. Poor correlation with chlorophyll at the active tillering stage suggests the disturbance of nutrient uptake by roots. Partial least squares path modeling (PLS-PM) results further supported that the pH_i_ directly affects grain yield and quality, as well as plant growth. The indirect effect via intervening fourth-week-variables was also substantial. Therefore, it is important to measure initial pH upon acid spill to estimate the risk to the paddy ecosystem. Information on the change in soil properties associated with acidity will also aid in predicting the yield and quality of grain to be harvested.

## 1. Introduction

With the increasing number and amount of chemicals consumed and distributed in various industrial sectors, there are concurrently growing concerns regarding chemical accidents resulting from human error, technical defects, or natural disasters. According to the environmental statistics provided by the National Institute of Chemical Safety, 607 cases of chemical accidents (e.g., spill, leakage, and explosion) have occurred during the last decade [1]. As part of protective measures, the Korean government has designated 97 industrial chemicals as accident preparedness substances (APS) owing to their acute toxicity, explosiveness, or high probability of chemical accident. Most APS are likely to cause severe damage to human health and the environment where chemical accidents occur. In response, special safety management protocols are deployed by public authorities to control the potential risks from these substances when handled, produced, transported, stored, or disposed of. In addition, the Ministry of Environment of Korea has established the data of human health risk potentially arising from APS to cope with emergency owing to chemical exposure. However, information on the impacts of these chemicals on the terrestrial ecosystem is scarce.

According to administrative statistics, there are 8298 km^2^ of paddy fields in Korea, accounting for ~52% of the total farmland [2]. Many paddy fields are located adjacent to industrial complexes or alongside chemical transport routes and are thus subject to damage by industrial chemicals. The paddy field is a unique system comprising various environmental matrices, e.g., flooded water, paddy soil, rice plants, and benthic biota. The paddy ecosystem provides various benefits, such as maintaining biodiversity by serving as habitats and food resources for a diverse biota, as well as rice production for human society [3].

There are several case reports of damage in paddy fields near the occurrence of chemical accidents. For example, environmental surveys were performed by public authorities to delineate the ecological impact of hydrofluoric acid gas leak from chemical plants in the Gumi industrial complex in 2012 [4,5]. They reported that crops and fruit on more than 200 hectares of farmlands were withered, and some 3200 livestock animals showed symptoms of nausea. Arable soils and crop plants were found to be highly contaminated with hydrofluoric acid. The Korean government designated the affected area as a special disaster zone, and later, approximately $38 million was paid in compensation to citizens and local businesses [5,6].

When a chemical accident occurs on farmland, stakeholders related to the accident want to minimize socio-economic problems by compensation and disposal of damaged crops through prompt investigations based on visual judgment by experts. In this process, disagreements among the parties may generate additional social costs. In order to minimize this problem, it is necessary to prepare scientific evidence through the establishment of terrestrial ecotoxicological data for substances with high risk of accidents. In Korea, terrestrial ecotoxicity data have been established thus far for only a few substances.

Oftentimes, the result of single species toxicity is used to interpret and estimate the ecological impact of chemical substances. For example, the reference concentrations (e.g., ecological soil screening level (Eco-SSL), environmental investigation levels (EILs), predicted no-effect concentration (PNEC), and hazardous concentration for x% of species (HC_x_)) are predicted from single species toxicity tests and employed for the terrestrial ecological risk assessment [7]. However, the result of these approaches can be misleading because the complexity of the ecosystem and interaction between ecosystem elements may be ignored [8]. Consequently, the integrated impact on the ecosystem cannot be accurately assessed. To overcome these limitations, ecological impact studies using mesocosms have been attempted so as to mimic various ecosystems, such as freshwater, marine, and terrestrial ecosystems [8,9,10]. As mentioned above, even though the occurrence frequency of chemical accidents along the paddy fields is increasingly high, ecotoxicity studies on paddy mesocosms are very limited.

Among the 97 chemicals in the APS list, HNO_3_ and H_2_SO_4_ are reported as the most concerned liquids of chemical accident, ranked first and fourth, respectively, according to the number of accidents between 2014 and 2018 in Korea [1]. Upon introduction into terrestrial environments, these strong acids are completely ionized and produce protons (H^+^) and oxyanions, such as NO_3_^−^ and SO_4_^2−^, both of which may function as nutrients for plant growth. Thus, an accidental spill of these acids into paddy fields can lead to an increment in nutrient supply, as well as toxic effects on terrestrial biota owing to corrosiveness of the acids. It is difficult to grasp these phenomena through short-term laboratory toxicity tests.

In this study, the effect of two strong acids (HNO_3_ and H_2_SO_4_) on the paddy ecosystem was investigated over a 20-week cropping period from transplanting to harvesting. The paddy mesocosm was designed using three representative species of different trophic levels; i.e., rice (*Oryza sativa*), golden apple snail (*Pomacea canaliculata*), and sludge worm (*Tubifex tubifex*) for the producer, consumer, and decomposer, respectively. Test chemicals were introduced at the level of observed PNEC or 50% hazardous concentration (HC_50_). Toxic endpoints of the test species (e.g., growth, mortality, population, and rice yield) were assessed at 4 and 20 weeks after chemical exposure. The causative relationship between the properties of paddy soil and rice growth and yield was also addressed based on the result of statistical analyses.

## 2. Materials and Methods

### 2.1. Mesocosm Setting

The mesocosm was set up in paddy fields (W × L = 35 m × 25 m) at the Korea University (KU) farm in Gyeonggi-do, Republic of Korea. Fifteen holes with a depth of 30 cm were dug at a spacing of 2 m × 3 m and the non-woven mat was laid on the bottom. Cylindrical rubber containers (top inner diameter =0.77 m, bottom inner diameter =0.65 m, and height =0.46 m with four bottom drains) were placed in each hole. Approximately 150 kg paddy soil was transferred into each container and then packed (bulk density of 1.2–1.3 g/cm^3^) by repeated flooding and drainage. Nine hills of 4-week-grown rice seedling (*O. sativa*), nine golden apple snails (*P. canaliculata*), and ninety sludge worms (*T. tubifex*) were introduced. Over the 22 weeks of the cropping period, common agronomic practices (e.g., intermittent flooding/drainage, 10 cm tillage, and 70 kg N ha^−1^) were performed as recommended by the Korea Rural Development Administration (RDA). Information regarding climate condition, management of paddy water, timings of test chemicals/test species addition, and life stage of rice are shown in Figure S1. Further information on test species (*O. sativa*, *P. canaliculata*, and *T. tubifex*) is also provided in the supplementary materials.

### 2.2. Test Chemicals

Two acids (HNO_3_ and H_2_SO_4_) were independently introduced into the paddy mesocosm one week after introducing the test animals (Figure S1) at two different levels; i.e., PNEC of *O. sativa* and HC_50_ of the terrestrial ecosystem. PNEC of *O. sativa* was calculated according to toxicity data from our laboratory: 12.5 and 20 mg kg^−1^ for HNO_3_ and H_2_SO_4_, respectively. The HC_50_ values were predicted by the USEPA SSD generator (ver. 1.0) using acute toxicity data: 1032 and 1849 mg kg^−1^ for HNO_3_ and H_2_SO_4_, respectively. The acute toxicity data (EC_50_) used for HC_50_ prediction were obtained from our previous study (Table S1). Methods used for the calculation of PNEC and HC_50_ are reported elsewhere [7,11]. All treatments, including the control, were conducted in triplicate. Hereafter, the treatments of HNO_3_ and H_2_SO_4_ at the level of PNEC and HC_50_ are referred to as HNO_3_-PNEC, HNO_3_-HC_50_, H_2_SO_4_-PNEC, and H_2_SO_4_-HC_50_.

### 2.3. Assessment of Impact on the Paddy Ecosystem

The pH of paddy soil (pH electrode at the ratio of 1:5) and chlorophyll content (SPAD-502 Plus, Konica Minolta, Osaka, Japan) of rice leaves were measured every week. Impact on test species were assessed twice at the 4th and 20th week after chemical exposure. The list of toxic endpoints employed in this study is summarized in Table S2. In the laboratory toxicity test, we found that the 4th week of exposure is appropriate to observe apparent response to acid exposure. After 20 weeks (i.e., the end of the rice cultivation period), rice endpoints such as grain yield and grain quality were also assessed.

#### 2.3.1. Short-Term Assessment at the 4th Week

In the 4th week after chemical exposure, three hills of the rice plant were removed from each treatment to collect growth data of rice, such as fresh weight (g) of shoot and height (cm) of shoot. The fresh weight of shoot per hill was measured using a balance and then divided by five to obtain the weight of each plant. The shoot height was obtained by measuring the length of the longest leaf from the shoot-root interface. The number of leaves damaged by chemical exposure was counted by visual inspection such as wilting, chlorosis, and rolling. The number of feeding-damaged leaves owing to the consumer (*P. canaliculata*) was obtained by counting the symptoms of feeding (e.g., cut leaves).

For *P. canaliculata*, survival rate (%), length of shell (cm), and weight (g) were measured. The survival rate was obtained by the percentage (%) of the number of collected surviving individuals in each treatment compared with that in the control. Shell length (cm) and weight (g) of the individuals were measured using a Vernier caliper and a balance, respectively. After measurement, the species were reintroduced into the system. To count the population of *T. tubifex*, 1.5 kg of rooting zone soil was sequentially wet sieved through 2000-, 500-, and 200-µm sieves to retrieve adult and juvenile worms from soil particles and plant residues. The worms retrieved by the 500-µm sieve were transferred to a Petri dish containing deionized water. The worms with visible gonads were counted as adult worms and the worms without the gonads were counted as juvenile worms.

#### 2.3.2. Long-Term Assessment at the 20th Week

In the 20th week, three hills of rice plants were randomly harvested from each system. The shoot weight (g) and plant height (cm) were measured for growth data as with the 4th week assessment. Upon harvesting the rice plant, the grain yield attribute was evaluated using the panicle number, grain number, and whole grain weight. The grain quality attribute was assessed using the 1000-grain weight and the percentage (%) of filled grains [12,13]. The panicle number per a plant was determined by counting the number of panicles in 10 randomly sampled plants in each treatment. The number of grains was counted using an automatic seed counter (CGOLDENWALL, Hangzhou, China). The whole grain weight (g) and 1000-grain weight (g) were measured using a balance after adjusting the moisture content to 14% [12]. For the % of filled grains, the number of grains sinking in 10% KCl solution (specific gravity =1.06 at 20 °C) was counted [13]. For *P. canaliculata* and *T. tubifex*, it was not possible to measure the 20-week toxicity endpoints because all test animals died by this time.

### 2.4. Paddy Soil Analysis at the 4th Week

Approximately 300 g of the topsoil (0–15 cm) was collected from each treatment and selected soil properties were determined as follows: soil pH (1:5 method) using an electrode (Orion 5 Star, Thermo Scientific, Beverly, MA, USA), soil organic carbon content using the chromic acid oxidation method (Walkley–Black method), NH_4_^+^, NO_2−_, NO_3−_, and available silicon using the colorimetric method, available silicate (SiO_4_^4−^) by the 1 N sodium acetate method, and available phosphorus (P_2_O_5_) using the Bray No. 1 method. Exchangeable cations (Ca^2+^, Mg^2+^, K^+^, and Na^+^) extracted by 1 N ammonium acetate were determined by inductively coupled plasma optical emission spectroscopy (730 Series, Agilent, Santa Clara, CA, USA).

### 2.5. Statistical Methods

Results of all measurements collected from treatments and the control were evaluated using one-way analysis of variance (ANOVA). For the data whose significance was verified (*F* < 0.05), post significance tests were performed using the pairwise Student’s *t*-test (α = 0.05). Pearson correlation analysis was performed between soil properties at the 4th week and the growth/productivity of rice (*O. sativa*). In addition, the correlation analysis was performed between rice productivity and chlorophyll content as a function of growth stage. These analyses were performed using SAS 9.4 (SAS Institute Inc., Cary, NC, USA). To determine the causative relationship between latent variables (Table S3), partial least squares path modeling (PLS-PM) analysis was performed using R (ver. 3.6.2, R Project for Statistical Computing). Details regarding the PLS-PM method are provided as supplementary material [14,15,16].

## 3. Results and Discussion

### 3.1. Acidity of Paddy Soils

**Figure 1:**
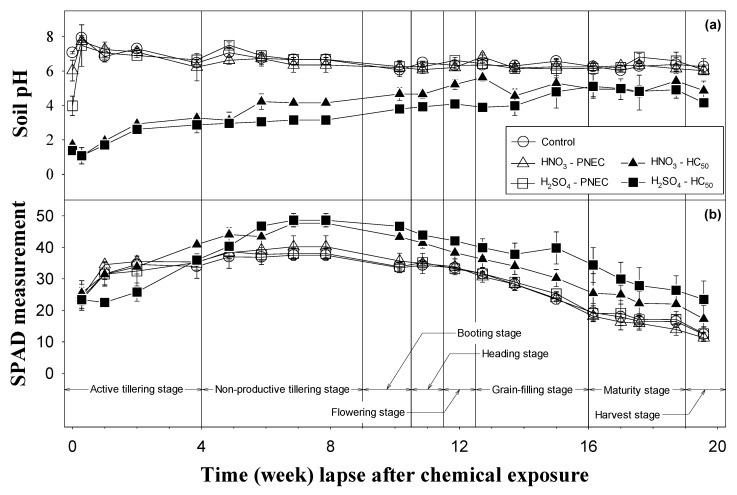
Variations in (**a**) pH of paddy soils and (**b**) chlorophyll content of rice (*Oryza sativa*) leaves in the four different treatments (HNO_3_-predicted no-effect concentration (PNEC), HNO_3_-hazardous concentration for 50% of species (HC_50_), H_2_SO_4_-PNEC, and H_2_SO_4_-HC_50_) during the 20-week experimental period. Data of the control are also shown for comparison. Growth stage of rice during the experimental is also shown along the *x*-axis.

Because most toxic effects of the two test chemicals (HNO_3_ and H_2_SO_4_) on the paddy ecosystem can be presumed to be caused by the activity of the hydrogen ion (H^+^), the paddy soil pH was monitored regularly as an important toxicity factor during the experimental period (Figure 1a). Immediately after the addition of acid, the pH values of all paddy soil treatments decreased significantly (*p* < 0.05). Note that the molar PNEC concentration of monoprotic HNO_3_ (=12.5 mg kg^−1^) and diprotic H_2_SO_4_ (=20 mg kg^−1^) is equally close to ~0.20 mmol kg^−1^. Therefore, H_2_SO_4_-PNEC is expected to result in twice the acidity of HNO_3_-PNEC. Indeed, as seen in Figure 1a, the pH of HNO_3_-PNEC and H_2_SO_4_-PNEC treatments upon chemical addition (=0th week) was 6.03 and 3.98, respectively. After two days, the pH of both PNEC treatments was statistically not different (α = 0.05) from that of the control, whereas the pH of the HC_50_ treatments remained extremely acidic (pH < 2). The soil pH of the HC_50_ treatments tended to increase slightly over time but was constantly lower than that of the control (α = 0.05) throughout the test period. Recall that the original paddy soil pH prior to the addition of acid was 6.26. The optimal soil pH for *O. sativa* cultivation is known to be around 6.0 ± 0.5 [17]. During the whole experimental period, the pH of the control and the two PNEC treatments was retained at around this optimal range. However, the pH of the two HC_50_ treatments was up to 5.63 units below the optimal range (Figure 1a) with a greater pH decrease found in the H_2_SO_4_-HC_50_ treatment.

### 3.2. Chlorophyll Content of Rice Leaves

Chlorophyll content (SPAD measurement) in the leaves of *O. sativa* is presented in Figure 1b with the lapse of time (i.e., weeks after chemical exposure) along with information on the growth phase of rice. For all treatments, including the control, the chlorophyll content increased gradually from the rooting stage to the tillering stage, and then exhibited the highest value between the non-productive tillering stage and booting stage during which the rice plant enters the reproductive phase. From the booting stage (i.e., ninth week), the chlorophyll content decreased gradually. A similar trend in chlorophyll was observed by Saberioon et al. [18] who reported that the SPAD measurements of leaves of *O. sativa* increases from the panicle initiation stage, tillering stage, to the middle booting stage and decreases from the late booting stage. Re-allocation of nutrients for grain production at the reproductive phase is known to reduce the chlorophyll content in the leaves after the booting stage [18].

In this study, the chlorophyll content of the PNEC treatments was not statistically different from that of the control (α = 0.05) over the experiment period, whereas that of the HC_50_ treatments varied widely. In particular, the chlorophyll content of the H_2_SO_4_-HC_50_ treatment was the lowest among treatments during the initial two weeks and then gradually increased above that of the control. Interestingly, a higher chlorophyll content was observed in both HNO_3_-HC_50_ and H_2_SO_4_-HC_50_ treatments starting from approximately the fifth week to the end of the experiment (Figure 1b).

### 3.3. Short-Term Impact Assessment

The feeding damage by *P. canaliculate* was found across four treatments and the control and the number of missing hills by feeding damage did not vary (*p* > 0.05). Thus, rice leaves (%) with feeding and wilting damage in Table 1 were calculated relative to surviving hill by this time.

#### 3.3.1. Growth of *O. Sativa* by the 4th Week

At the fourth week after chemical exposure, three hills of rice plants were randomly sampled from each mesocosm. No visible symptoms (i.e., chlorosis, leaf curling, and withering) were observed in rice leaves in the control; however, the impact of acids was apparent in the treatments. Visual inspection revealed that the canopies of *O. sativa* were damaged in the two HC_50_ treatments compared with that in the control. In the HNO_3_-PNEC treatment, shoots of several hills disappeared with or without leaving remnants. We judged these to be eaten by *P. canaliculata*. Across the four treatments, residues of withered leaves were detected. These leaves were judged as wilting damage owing to the acids.

The fourth week rice data are presented in Table 1, including the chlorophyll content, fresh weight of the shoot, plant height, the % of leaves with feeding damage, and the % of leaves with wilting damage. Firstly, the chlorophyll content in all treatments, except for HNO_3_-HC_50_, was not significantly different from that of the control. The fresh weight of the shoot and the plant height were significantly lower in the H_2_SO_4_-HC_50_ treatment. Growth of rice was slightly inhibited in the HNO_3_-HC_50_ treatment; however, the magnitude was statistically insignificant. The feeding damage (%) observed in the two PNEC treatments was not statistically different from that of the control. However, it was much lower in the two HC_50_ treatments which implies that the activity of the consumer (*P. canaliculata*) is possibly impeded by the high concentration (HC_50_) of acids. Leaves with wilting damage were observed in all four treatments in the order of HNO_3_-HC_50_ ≥ H_2_SO_4_-HC_50_ >> HNO_3_-PNEC ≥ H_2_SO_4_-PNEC treatments. Addition of HNO_3_ and H_2_SO_4_ increases the acidity of the paddy system and the corrosiveness of H^+^ seems to have influenced the initial growth of rice plants [19]. In summary, the fresh weight, plant height, and feeding damage were greatly affected by the H_2_SO_4_-HC_50_ treatment. At four weeks, wilting damage was detected in all treatments, whereas variation in chlorophyll content was not significant between the treatments, except for HNO_3_-HC_50_ and the control.

#### 3.3.2. Growth of *P. Canaliculata*

At the fourth week, all nine *P. canaliculata* initially added to the paddy systems were recovered from the PNEC treatments (i.e., 100% survival rate), but none were recovered from the two HC_50_ treatments (i.e., 0% survival rate), regardless of the type of acid (Table 1). The pH tolerance of snail species belonging to the same class (Gastropoda) as *P. canaliculata*, has been reported to be between 5.5 and 9.5 [20]. The extreme acidity (e.g., pH < 3.8 during the first to fourth week; Figure 1a) developed in the HC_50_ treatments seems detrimental to *P. canaliculata*, leading to high fatality. Therefore, the low feeding damage in rice leaves observed in the two HC_50_ treatments is most likely owing to the fatality of *P. canaliculata* because of the addition of a high dose of acid.

For the shell length and weight, the measured values from the H_2_SO_4_-PNEC treatment were significantly lower than those of the control, whereas the similar impact was not apparent in the HNO_3_-PNEC treatment (Table 1) most likely owing to the greater acidity of the former (Figure 1a). It has been established that acidic pH (<3.5–4.0) is a critical limiting factor for the growth of snail species, inhibiting construction of the shell and maintenance of mollusks [21].

#### 3.3.3. Population of *T. Tubifex*

Similar to *P. canaliculata*, none of the *T. tubifex* were recovered from the HC_50_ treatments of both acids (i.e., 0% survival rate) (Table 1). For *T. tubifex*, the pH tolerance range is known as 6.0–11.0 and the exposure to low pH (<4.2) is fatal for most species [22]. In this study, the number of *T. tubifex* recovered from the two PNEC treatments was found to be lower than that of the control. For example, the number of adults recovered from the HNO_3_-PNEC and H_2_SO_4_-PNEC treatments was reduced by 30.2% and 46.5%, respectively. It is worthwhile to note that juveniles were recovered from the two PNEC treatments which indicates the occurrence of reproduction within four weeks. However, the number of juveniles recovered from the two PNEC treatment was greatly reduced; that is, 82.9% and 84.0% reduction in juvenile population in the HNO_3_-PNEC and H_2_SO_4_-PNEC treatments, respectively. Under the experimental setting of this study, the reproduction of *T. tubifex* seems to be more affected than the mortality of the adults.

### 3.4. Chemical Properties of Paddy Soil in the 4th Week

Soil chemical properties related to rice growth are presented in Table S4, including organic carbon (SOC), inorganic nitrogen (NH_4_^+^, NO_2_^−^, and NO_3_^−^), available phosphorus (Av-P), available silicate (Av-Si), and exchangeable potassium (K^+^). The level of SOC and Av-P did not vary and remained within the typical range for rice cultivation [23,24]. Rice is a representative Si-accumulating plant [25]. The level of Av-Si was statically low in the two HC_50_ treatments. However, the range of the element (1.33–1.91 mmol kg^−1^) was close to the optimum value (1.28–1.78 mmol kg^−1^) recommended by Paye et al. [25]. Similarly, the level of K^+^ in the paddy field (2.34–3.29 mmol kg^−1^) was not limited for rice cultivation (1.7–2.1 mmol kg^−1^) [23].

Nitrogen (N) is an essential nutrient for plant growth, development, and reproduction [26]. Both deficiency and oversupply of the element can cause nutritional problems affecting rice growth and productivity. N-deficient rice exhibits inhibition of tillering, narrow and short leaves, thin stems, and an earlier maturity stage than normal rice. In contrast, excessive N results in overgrowth of shoots and easy lodging. For harvested rice, N-oversupply may lead to a deterioration in grain quality [27,28]. In this study, the level of NH_4_^+^ in the two HC_50_ treatments was approximately twice that of the control, most likely owing to the enhanced ammonification process in the acidic soil [29]. As expected, the level of NO_3_^−^ was the highest in the HNO_3_-HC_50_ treatment (23.4 mmol N kg^−1^ which is approximately 8.4-fold that of the control). The NO_3_^−^ in the H_2_SO_4_-HC_50_ treatment was slightly high; however, the difference was not significant. The NO_2_^−^ level was negligible in this study. Note that initially, N fertilizer was applied at the ratio of 70 kg N ha^−1^ in all the paddy systems. At the fourth week, inorganic N levels (as unit of N kg ha^−1^ by summing NH_4_^+^ and NO_3_^−^) of the HNO_3_-PNEC, HNO_3_-HC_50_, H_2_SO_4_-PNEC, and H_2_SO_4_-HC_50_ treatments were 41.1, 493.4, 35.7, and 58.5 N kg ha^−1^, respectively. The level in the control was 31.9 N kg ha^−1^. Therefore, the N level of HNO_3_-HC_50_ and H_2_SO_4_-HC_50_ treatments are 15.2-fold and 1.8-fold the level of the control, respectively. Reduction in the N level (~38.1 N kg ha^−1^) in the control at the fourth week relative to the initial application level appears to be because of N mass loss by plant uptake and downward leaching.

### 3.5. Long-Term Impact Assessment

The one-way ANOVA result indicated the significant difference (*p* < 0.05) in the chlorophyll content, grain number, whole grain weight, 1000-grain weight, and the % of filled grain between the treatments. However, differences in the fresh weight, plant height, and panicle number were not significant (*p* > 0.05).

#### 3.5.1. Growth and Yield of *O. Sativa* at the 20th Week

The canopies of rice plants in all treatments were well developed by the 20th week such that the difference was indiscernible by visual observation. The measurement data of growth and yield of *O. sativa* at the 20th week are presented in Table 2. Firstly, chlorophyll content at the 20th week was much lower than that at the fourth week and did not vary greatly across treatments, except for the H_2_SO_4_-HC_50_ treatment. Similarly, the fresh weight and shoot height of the four treatments were not statistically different from those of the control. As seen in Table 2, the fresh weight of rice grown in HNO_3_-HC_50_ and H_2_SO_4_-HC_50_ treatments was 14% and 25%, respectively, greater than that of the control, even though the difference was statistically insignificant. Therefore, it can be stated that the inhibitory effect of acids on initial rice growth becomes moderately attenuated with the lapse of time, probably because of the pH neutralization capacity of the paddy system.

Secondly, the grain yield trait was characterized by measuring three yield attributes including the panicle number, whole grain number, and whole grain weight, whereas 1000-grain weight and filled grain (%) were measured as grain quality traits (Table 2). For the harvested rice, the three endpoints associated with grain yield trait were no less than those of the control. For example, increments of 47.9% in the panicle number, 73.6% in whole grain number, and 47.4% in whole grain weight were observed in the HNO_3_-HC_50_ treatment. The data of the two PNEC treatments were not significantly different from that of the control. In contrast, the trend of two endpoints associated with grain quality differed from that of grain yield. Both 1000-grain weight and filled grain (%) were lower in the two HC_50_ treatments. In particular, the degree of filled grains (%) of the HNO_3_-HC_50_ and H_2_SO_4_-HC_50_ treatments were 21.5% and 39.7%, respectively, both of which are lower than that of the control. It was found that in both HC_50_ treatments, the grain yield trait is better, whereas the grain quality trait is lower than that of the control. According to Panda et al. [27], rice growth and grain quality, such as panicle weight, dry biomass, and filled grain (%), tend to increase with increasing N application up to 80 kg N ha^−1^, but decreases at 120 kg N ha^−1^. Therefore, in this study, acidic pH in the HC_50_ treatments most likely deteriorate the grain quality at the 20th week. Moreover, the oversupply of N in HNO_3_-HC_50_ also affects the grain quality.

#### 3.5.2. Fatality of *P. Canaliculata* and *T. Tubifex*

In the 20th week, none of the *P. canaliculata* and *T. tubifex* were recovered from all treatments including the control (i.e., 0% survival rate). It is understood that the fatality of the test animals is not solely caused by the toxicity of the test chemicals. Note that this mesocosm study was conducted under the common Korea rice farming practice in which two intermittent drainage events are employed at the 6th and 14th week (Figure S1). It is believed that the desiccated paddy environment during the drainage periods appears to be fatal to the survival of the test animals. In real-life, the test animals can survive in paddy fields even during a drought period by self-burial under soil or migrating to nearby wet spots (e.g., farm waterway) [30]. However, the test animals could hardly survive in this study setup because the paddy mesocosm was disconnected from the external waterway and the paddy soil pores became desiccated during the drainage period. It is reasonable to state that the design of the paddy system does not adequately imitate the environment, interfering with the natural behavior of the test animals in their original ecological settings. Therefore, in the case of the closed paddy system, the *P. canaliculata* and *T. tubifex* are limitedly applicable for evaluating the impact of test chemicals under flooded paddy condition.

### 3.6. Statistical Analysis

#### 3.6.1. Pearson Correlation

##### Correlation between Rice Productivity and Soil Properties at the 4th Week

Among the soil data for the fourth week (Table S4), the variation in three variables (pH, NH_4_^+^, and NO_3_^−^) was meaningful across treatments and the control. The Pearson correlation analysis between these variables and the 20th week endpoints (e.g., plant growth, grain yield, and grain quality) was performed as shown in Table 3. The pH exhibited negative correlations with all endpoints associated with plant growth and grain yield, whereas positive correlation with grain quality; that is, chlorophyll (*r* = −0.69, *p* = 0.006), shoot height (*r* = −0.41, *p* = 0.14), fresh weight (*r* = −0.59, *p* = 0.03), panicle number (*r* = −0.67, *p* = 0.008), grain number (*r* = −0.74, *p* = 0.002), whole grain weight (*r* = −0.75, *p* = 0.002), 1000-grain weight (*r* = 0.62, *p* = 0.02), and filled grain (%) (*r* = 0.67, *p* = 0.009). Conversely, the result for inorganic nitrogen (NH_4_^+^ and NO_3_^−^) was the opposite. In general, the concentration of both NH_4_^+^ and NO_3_^−^ was positively correlated with plant growth (chlorophyll, shoot height, and fresh weight) and grain yield (panicle number, grain number, and whole grain weight), whereas it was negatively correlated with grain quality (1000-grain weight and filled grain (%)). Negative correlation between the level of inorganic N and grain quality supports our speculation that oversupply of N into the paddy field deteriorates the quality of harvested rice. Correlation results indicate that paddy soil properties, such as initial pH and nitrogen level, can be used as indicators for predicting the growth and productivity of rice.

##### Correlation between Rice Productivity and Chlorophyll Content as a Function of Time

Grain yield and quality were correlated with the chlorophyll content in the rice leaf as a function of life in Table S5. For the active tillering stage (~fourth week), chlorophyll content was not well correlated (−0.18 ≤ *r* ≤ 0.28) with any of the endpoints of rice productivity. From the non-productive tillering stage (fifth week~) until the ripening phase (~20th week), it was fairly well correlated with all endpoints; positive correlation with grain yield parameters (0.62 ≤ *r* ≤ 0.79) and negative correlation with grain quality parameters (−0.45 ≤ *r* ≤ −0.90).

In general, it is well understood that the chlorophyll content in rice is a reliable indicator for predicting grain N status and productivity [31]. Poor correlation was observed only for the active tillering stage, and therefore indicates that uptake of nutrients (e.g., nitrogen) by the root, and thus, the formation of chlorophyll by the leaf are disturbed by the addition of acid during the initial growth stage (~fourth week). Fair correlation after the fifth week and later growth stage suggests that nutrient uptake and chlorophyll formation are gradually restored to normal levels with the passage of time. Therefore, in the case of rice grown in acids-impacted paddy fields, initial chlorophyll content may be appropriate to be used as an indicator for long-term endpoints (e.g., rice productivity).

#### 3.6.2. Causative Relationship between Latent Variables

**Figure 2:**
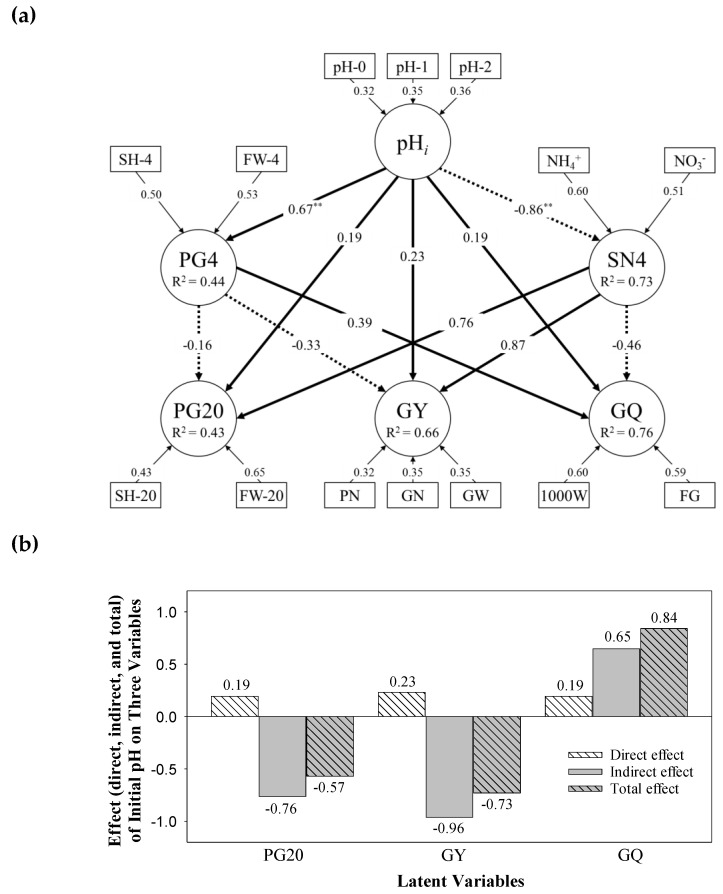
Result of partial least squares path modeling (PLS-PM). (**a**) Unidirectional cause–effect between six latent variables are shown as an arrow with path coefficients on it (solid and dotted arrows denote positive and negative effects, respectively). The value above the arrow in the outer model represents the weight of the measured variable. The number of asterisks indicates the significance level (^**^ *p* < 0.01). *R*^2^ indicates the determination coefficient of each latent variable in the inner model. (**b**) Direct, indirect, and total effects of initial paddy soil pH (pH*_i_*) on three latent variables related to rice growth and productivity (PG20, GY, and GQ).

It was apparent that the early condition of paddy soil and rice growth are related to rice productivity at the 20th week. The result of PLS-PM analysis is presented in Figure 2 as the structural equation models comprising six latent variables (pH*_i_*, SN4, PG4, PG20, GY, and GQ; refer to Table S3 for acronyms). The overall goodness of fit index was 0.731.

Firstly, the outer model in Figure 2a describes the relations among the measured variables (rectangles) and the corresponding latent variables (circles). The number on the arrow is the weight of the measured variables on the latent variable. For example, the latent variable pH*_i_* was defined by 32–36% weight of three measured pH values. The result of the validity test is reported in Table S3. Dillon–Goldstein’s Rho value for the equations (0.828–0.987) were above the suggested threshold value of 0.7, indicating sufficient internal consistency. Furthermore, the AVE value for all latent variables were above the recommended threshold of 0.5. These results demonstrate adequate reliability and validity of the constructed equation for latent variables.

The inner model describes the unidirectional relationship between latent variables (circles) with an arrow and path coefficient on it. The solid and dotted arrows indicate the positive and negative relations, respectively; thus, the negative value of the path coefficient is on the dotted arrow. The latent variable pH*_i_* exerted the greatest effect on PG4 and SN4, with a path coefficient of 0.67 and −0.89, respectively, whereas its effect on PG20, GY, and GQ was relatively small (0.19–0.23). The impact of SN4 on PG20, GY, and GQ was greater than that of PG4 on these three variables, as determined by the magnitude of the path coefficient for the given pairwise relations, even though the sign of the path coefficient was opposite. For example, the effect of PG4 on GY is –0.33, whereas the effect of SN4 on GY is 0.86.

Note also that the indirect effect occurs between pH*_i_* and GY through intervening variables such as pH*_i_*→ PG4 → GY and pH*_i_*→ SN4 → GY. The indirect path coefficient of these relations can be calculated as 0.67 × (−0.33) = −0.22 for the former and (−0.86) × 0.86 = −0.74 for the latter. Therefore, the total effect of pH*_i_* on GY, the sum of direct and indirect effects, will be 0.23 + (−0.22) + (−0.74) = 0.73. Figure 2b shows the magnitude of direct and indirect effects of pH*_i_* on PG20, GY, and GQ. Both PG20 and GY showed a similar trend. The positive direct effect was offset by a greater negative effect; thus, the variable pH*_i_* comes to have a negative total effect on both PG20 (−0.57) and GY (−0.73). In contrast, the relation between pH*_i_* and GQ, indirect effect (0.65), was additive for the direct effect (0.19); thus, resulting in a positive total effect (0.84). As noted earlier, the direct effect of initial pH (pH*_i_*) on the 20th week endpoints (PG20, GY, and GQ) was small. However, the variable pH*_i_* can indirectly influence these three variables to a great extent via PG4 an SN4 (Figure 2b).

## 4. Conclusions

Results of this study demonstrate that growth of *O. sativa*, mortality and growth of *P. canaliculata*, and population of *T. tubifex* are the meaningful endpoints for assessing short-term (e.g., four weeks) effects of acid exposure, but not for 20 weeks. Mortality of the test animals can be used as endpoints to assess short-term impact with exposure at the HC_50_ level. In addition, the growth of *P. canaliculata* and population of *T. tubifex* can be appropriate endpoints for evaluating impacts at the PNEC level. The sensitivity of the test species for the two strong acids was in the order of *T. tubifex* > *P. canaliculata* > *O. sativa*. However, *P. canaliculata* and *T. tubifex* were inadequate for assessing long-term toxicity in this study because their natural behavior was interfered with in our paddy mesocosm setup. It shows the limitations of a closed mesocosm system that fails to mimic the natural ecosystem. Nonetheless, we could evaluate the short-term effects of both acids on a community level by this paddy mesocosm. Through further investigation involving microbial community and nutrient cycling in an improved mesocosm design, the effects of chemicals on paddy ecosystem, beyond the community level, can be assessed.

In short, the introduction of the acids to paddy fields initially decreases the soil pH, which disturbs the balance of soil nutrients and the formation of chlorophyll. The alteration subsequently affects plant growth and grain yield/quality attributes. A causative relationship was statistically apparent between initial pH and rice growth and productivity upon harvest. These variables are also indirectly related through intervening variables, such as early growth response and soil nitrogen level. Information of initial acidity and associated soil property change can aid in predicting the yield and quality of grain to be harvested. Therefore, this study highlights the importance of measuring pH of paddy fields after the introduction of acid chemicals. Furthermore, in order to establish the resilience of the paddy ecosystem against acid spill accidents, increasing the pH-buffering capacity of paddy systems should be considered foremost.

## Figures and Tables

**Table 1:** Results ^1^ of measured endpoints of three test species (*O. sativa*, *Pomacea canaliculata*, and *Tubifex tubifex*) at the 4th week.

Species	Endpoint	Control	Treatment			
			HNO_3_		H_2_SO_4_	
			PNEC ^2^	HC_50_^3^	PNEC ^2^	HC_50_^3^
Rice (*O. sativa*)	Chlorophyll content	33.9 ± 3.76 a	35.4 ± 2.58 a	40.9 ± 0.89 b	35.2 ± 1.01 a	36.0 ± 1.40 a
	Fresh weight of shoot (g)	1.90 ± 0.55 a	1.89 ± 0.60 a	1.13 ± 0.62 ab	1.63 ± 0.36 a	0.32 ± 0.16 b
	Plant height (cm)	38.0 ± 2.91 a	33.7 ± 5.15 a	32.6 ± 3.73 ab	36.0 ± 0.84 a	26.5 ± 3.21 b
	Feeding damage (%)	11.3 ± 5.17 a	10.9 ± 2.56 a	7.93 ± 3.63 ab	13.0 ± 2.91 a	2.56 ± 4.44 b
	Wilting damage (%)	0 a	16.1 ± 5.31 bc	31.1 ± 11.6 d	12.1 ± 3.02 b	25.8 ± 5.42 cd
Golden apple snail (*P. canaliculata*)	Survival rate (%)	100 a	100 a	0 b	100 a	0 b
	Shell length (mm)	26.9 ± 1.79 a	26.3 ± 1.07 a	ND ^4^	24.9 ± 1.37 b	ND
	Weight (mg)	5.84 ± 1.09 a	5.31 ± 0.82 a	ND	4.33 ± 0.72 b	ND
Sludge worm (*T. tubifex*)	Number of adults	9.56 ± 0.53 a	6.67 ± 5.00 b	0 c	5.11 ± 4.23 b	0 c
	Number of juveniles	20.8 ± 29.0 a	3.56 ± 6.31 b	0 b	3.33 ± 5.00 b	0 b

^1^ Mean ± standard deviation. The different letters indicate significant differences among groups at *p* < 0.05. ^2^ Predicted no effect concentration of *O. sativa*; 12.5 and 20 mg kg^−1^ for HNO_3_ and H_2_SO_4_ treatments, respectively. ^3^ Hazardous concentration for 50% of terrestrial species; 1032 and 1849 mg kg^−1^ for HNO_3_ and H_2_SO_4_ treatments, respectively. ^4^ Not determined.

**Table 2:** Results ^1^ of rice growth and yield/quality attributes of the harvested rice measured at the 20th week.

Type	Endpoint	Control	Treatment			
			HNO_3_		H_2_SO_4_	
			PNEC ^2^	HC_50_^3^	PNEC ^2^	HC_50_^3^
Plant growth	Chlorophyll content	12.3 ± 2.17 a	11.3 ± 1.20 a	17.3 ± 4.28 ab	12.6 ± 2.25 a	23.5 ± 5.87 b
	Fresh weight (g)	29.5 ± 6.46 a	28.4 ± 12.0 a	36.8 ± 12.4 a	23.5 ± 6.22 a	33.7 ± 15.4 a
	Plant height (cm)	83.6 ± 4.79 a	88.4 ± 4.74 a	88.7 ± 10.2 a	86.3 ± 5.51 a	88.4 ± 6.15 a
Grain yield	Panicle number	21.9 ± 5.75 a	23.5 ± 6.62 a	32.4 ± 8.24 a	20.2 ± 4.22 a	27.5 ± 8.77 a
	Grain number	11,046 ± 2539 a	11,937 ± 1396 a	19,178 ± 4644 b	10,532 ± 973 a	15,110 ± 277 ab
	Whole grain weight (g)	301 ± 63.3 ab	316 ± 29.5 ab	443 ± 92.6 c	281 ± 21.4 a	390 ± 8.49 bc
Grain quality	1000-grain weight (g)	22.7 ± 0.18 a	22.7 ± 0.70 a	19.8 ± 1.21 b	22.4 ± 0.56 a	22.1 ± 0.08 a
	Filled grain (%)	94.7 ± 2.08 a	93.8 ± 1.63 a	74.3 ± 6.71 ab	93.0 ± 3.17 a	57.1 ± 38.0 b

^1^ Mean ± standard deviation. The different letters indicate significant differences among groups at *p* < 0.05. ^2^ Predicted no effect concentration of *O. sativa*; 12.5 and 20 mg kg^−1^ for HNO_3_ and H_2_SO_4_ treatments, respectively. ^3^ Hazardous concentration for 50% of terrestrial species; 1032 and 1849 mg kg^−1^ for HNO_3_ and H_2_SO_4_ treatments, respectively.

**Table 3:** Pearson correlation coefficient ^1^ (*r*) between selected soil properties at the 4th week and the endpoint of rice at the 20th week.

	Endpoint	Plant Growth			Grain Yield			Grain Quality	
Soil Property		Chlorophyll	Shoot Height	Fresh Weight	Panicle Number	Grain Number	Whole Grain Weight	1000-Grain Weight	Filled Grain Percentage
pH		−0.69 ^**^	−0.41	−0.59 ^*^	−0.67 ^**^	−0.74 ^**^	−0.75 ^**^	0.62 ^*^	0.67 ^**^
NH_4_^+^		0.65 ^*^	0.57 ^*^	0.64 ^*^	0.63 ^*^	0.68 ^**^	0.70 ^**^	−0.51	−0.65 ^*^
NO_3−_		0.15	0.39	0.52	0.70 ^**^	0.73 ^**^	0.68 ^**^	−0.81 ^***^	−0.29

^1^ The number of asterisks indicates the significance level of correlation: ^*^ *p* ≤ 0.05, ^**^ *p* ≤ 0.01, and ^***^ *p* ≤ 0.001. Correlations without asterisk are not statistically significant (*p* > 0.05).

## Supplementary Materials

The following are available online at https://www.mdpi.com/1660-4601/17/14/5244/s1, Materials and Methods: Description of partial least squares path modeling, Table S1: Acute toxicities of HNO_3_ and H_2_SO_4_ to five terrestrial species, Table S2: List of toxic endpoints for test species measured at the 4th and 20th week, Table S3: List of six latent variables and their corresponding measured variables used in this model, Table S4: Results of paddy soil analysis at the 4th week, Table S5: Pearson correlation coefficient between rice productivity and chlorophyll content as a function of growth stage, Figure S1: Timewise condition during the experimental period.

## References

[1] National Institute of Chemical Safety (NICS) Comprehensive Chemical Information System. http://icis.me.go.kr/.

[2] Korean Statistical Information Service (KOSIS) (2020). 2019 Agricultural Area Survey.

[3] Bang H.-S., Han M.-S., Na Y.-E., Kim M.-H., Kang K.-K., Lee J.-T., Lee D.-B., Nakano S., Yahara T., Nakashizuka T. (2012). Biodiversity of inhabitants of animals and vascular plants In Korean paddy fields ecosystem. The Biodiversity Observation Network in the Asia-Pacific Region.

[4] An J., Lee H.A., Lee J., Yoon H.-O. (2015). Fluorine distribution in soil in the vicinity of an accidental spillage of hydrofluoric acid in Korea. Chemosphere.

[5] Gumi-city (2013). Hydrofluoric Acid Changes Gumi: Hydrofluoric Acid Leakage Accident White Paper.

[6] Kim Y., Jang H., Choi Y., Sohn H.-G. (2015). Disaster classification for optimal disaster response in Korea. J. Korean Soc. Hazard. Mitig..

[7] Frampton G.K., Jansch S., Scott-Fordsmand J.J., Rombke J., Van den Brink P.J. (2006). Effects of pesticides on soil invertebrates in laboratory studies: A review and analysis using species sensitivity distributions. Environ. Toxicol. Chem..

[8] Van den Brink P.J., Tarazona J.V., Solomon K.R., Knacker T., Van den Brink N.W., Brock T.C.M., Hoogland J.P. (2005). The use of terrestrial and aquatic microcosms and mesocosms for the ecological risk assessment of veterinary medicinal products. Environ. Toxicol. Chem..

[9] Grantham T.E., Canedo-Arguelles M., Perree I., Rieradevall M., Prat N. (2012). A mesocosm approach for detecting stream invertebrate community responses to treated wastewater effluent. Environ. Pollut..

[10] Wetzel M.A., Wahrendorf D.-S., Von der Ohe P.C. (2013). Sediment pollution in the Elbe estuary and its potential toxicity at different trophic levels. Sci. Total Environ..

[11] European Chemicals Agency (2008). Guidance on Information Requirements and Chemical Safety Assessment—Chapter R.10: Characterisation of Dose [Concentration]-Response for Environment.

[12] Zhang Z., Zhang S., Yang J., Zhang J. (2008). Yield, grain quality and water use efficiency of rice under non-flooded mulching cultivation. Field Crop. Res..

[13] Liu L., Chen T., Wang Z., Zhang H., Yang J., Zhang J. (2013). Combination of site-specific nitrogen management and alternate wetting and drying irrigation increases grain yield and nitrogen and water use efficiency in super rice. Field Crop. Res..

[14] Sanchez G. PLS Path Modeling with R.

[15] Nitzl C., Roldan J.L., Cepeda G. (2016). Mediation analysis in partial least squares path modeling: Helping researchers discuss more sophisticated models. Ind. Manag. Data Syst..

[16] Henseler J., Sarstedt M. (2013). Goodness-of-fit indices for partial least squares path modeling. Comput. Stat..

[17] Gnanamanickam S.S., Candole B.L., Mew T.W. (1992). Influence of soil factors and cultural practice on biological control of sheath blight of rice with antagonistic bacteria. Plant. Soil.

[18] Saberioon M.M., Amin M.S.M., Anuar A.R., Gholizadeh A., Wayayok A., Khairunniza-Bejo S. (2014). Assessment of rice leaf chlorophyll content using visible bands at different growth stages at both the leaf and canopy scale. Int. J. Appl. Earth Obs. Geoinf..

[19] Menzies N.W., Rengel Z. (2003). Toxic elements in acid soils: Chemistry and measurement. Handbook of Soil Acidity.

[20] Bernatis J.L., Mcgaw I.J., Cross C.L. (2016). Abiotic tolerances in different life stages of apple snails *Pomacea canaliculata* and *Pomacea maculata* and the implications for distribution. J. Shellfish Res..

[21] Ramakrishnan V. (2007). Salinity, pH, Temperature, Desiccation and Hypoxia Tolerance in the Invasive Freshwater Apple Snail *Pomacea Insularum*. Ph.D. Thesis.

[22] Whitley L.S. (1968). The resistance of tubificid worms to three common pollutants. Hydrobiologia.

[23] Dobermann A., Cassman K.G., Mamaril C.P., Sheehy J.E. (1998). Management of phosphorus, potassium, and sulfur in intensive, irrigated lowland rice. Field Crop. Res..

[24] Minasny B., Hong S.Y., Hartemink A.E., Kim Y.H., Kang S.S. (2016). Soil pH increase under paddy in South Korea between 2000 and 2012. Agric. Ecosyst. Environ..

[25] Paye W., Tubana B., Harrell D., Babu T., Kanke Y., Datnoff L. (2018). Determination of critical soil silicon levels for rice production in Louisiana using different extraction procedures. Commun. Soil Sci. Plant. Anal..

[26] Hawkesford M., Horst W., Kichey T., Lambers H., Schjoerring J., Moller I.S., White P., Marschner P. (2012). Chapter 6—Functions of macronutrients. Marschner’s Mineral Nutrition of Higher Plants.

[27] Panda B.B., Sharma S., Mohapatra P.K., Das A. (2012). Application of excess nitrogen, phosphorus, and potassium fertilizers leads to lowering of grain iron content in high-yielding tropical rice. Commun. Soil Sci. Plant. Anal..

[28] Weil R.R., Brady N.C., Weil R.R., Brady N.C. (2017). Chapter 13—Nitrogen and sulfur economy of soils. The Nature and Properties of Soils.

[29] De Vries W., Breeuwsma A. (1987). The relation between soil acidification and element cycling. Water Air Soil Pollut..

[30] Yusa Y., Wada T., Takahashi S. (2006). Effects of dormant duration, body size, self-burial and water condition on the long-term survival of the apple snail, *Pomacea canaliculata* (Gastropoda: Ampullariidae). Appl. Entomol. Zool..

[31] Ramesh K., Chandrasekaran B., Balasubramanian T.N., Bangarusamy U., Sivasamy R., Sankaran N. (2002). Chlorophyll dynamics in rice (*Oryza sativa*) before and after flowering based on SPAD (chlorophyll) meter monitoring and its relation with grain yield. J. Agron. Crop. Sci..

