# Supplementary material for: Effect of HNO3 and H2SO4 on the Paddy Ecosystem: A Mesocosm Study with Exposure at PNEC and HC50 Levels
Source: Int J Environ Res Public Health. 2020 Jul 21;17(14):5244. doi: 10.3390/ijerph17145244 (PMC7400452; doi:10.3390/ijerph17145244)
Supplement: Supplementary file 1 [file ijerph-17-05244-s001.pdf]

International Journal of
Environmental Research and Public Health

MDPI

Supplementary Materials

Effect of HNO_3 and H_2SO_4 on the Paddy Ecosystem: A Mesocosm Study with Exposure at PNEC and HC_{50} Levels

Minseok Park, Wonjae Hwang, Jino Son, June Wee, Kijong Cho and Seunghun Hyun *

Department of Environmental Science and Ecological Engineering, Korea University, Seoul, 02841 KOREA; asithinkyou@korea.ac.kr (M.P.), hwj0145@korea.ac.kr (W.H), rogix2001@korea.ac.kr (J.S.), dnlwns@korea.ac.kr (J.W.), kjcho@korea.ac.kr (K.C.), soilhyun@korea.ac.kr (S.H.)

* Correspondence: soilhyun@korea.ac.kr; Tel.: +82-2-3290-3068

List of contents: Materials and Methods. Test species (Supplementary information for 2.1. Mesocosm setting). Partial least squares path modeling (PLS-PM) (Supplementary information for 2.5. Statistical method). **Table S1.** Acute toxicities (EC_{50}, mg kg^{-1}) of HNO_3 and H_2SO_4 to five terrestrial species (*Oryza sativa*, *Raphanus sativus*, *Pinus densiflora*, *Paronychiurus kimi*, and *Eisenia fetida*). **Table S2.** List of toxic endpoints for test species measured at the 4th and 20th week. **Table S3.** List of six latent variables and their corresponding measured variables used in this model. Latent variables are defined by the weighted sum of measured variables as given below. **Table S4.** Results of paddy soil analysis at the 4th week. **Table S5.** Pearson correlation coefficient (*r*) between rice productivity and chlorophyll content as a function of growth stage. **Fig. S1.** Timewise condition during the 20–week experimental period (a) precipitation and temperature, (b) the level of paddy water along with the timing of test species and test chemicals introduction, and (c) life stage of rice (*Oryza sativa*) cultivation during the cropping season (from germination to harvest).

1. Materials and Methods

1.1. Test species

For test species of paddy mesocosm, rice (*Oryza sativa*), golden apple snail (*Pomacea canaliculata*), and sludge worm (*Tubifex tubifex*) were used in this study. The rice variety (*O. sativa var. misomi*) was developed by the Korea RDA in 2013 and is currently cultivated nation-wide across the Korea Peninsula owing to its resistance to blast disease. The seeds were obtained from KU farm. For each treatment, nine hills of 4–week-old seedlings grown in the nursery were transplanted at a spacing of 21 cm × 21 cm. Each hill had five rice seedlings. Golden apple snails (*P. canaliculata*) was purchased from the Buyong golden apple snail farm (Gyeonggi-do, Korea). Nine *P. canaliculata*, with an average weight of 2.39 ± 0.64 g and an average shell length of 20.36 ± 1.95 mm, were placed on the top of paddy soil. Sludge worms (*T. tubifex*) were purchased from Alpha Fish, Korea (http://alphafish.co.kr/). Ninety adult *T. tubifex* with visible gonads were added at a 5-cm soil depth. Both test animals were introduced on the 5th day after transplanting to avoid seedling damage by the snails.

Three test species, *O. sativa*, *P. canaliculata*, and *T. tubifex*, represent different trophic levels, producer, consumer, and decomposer, respectively. *O. sativa* is the most cultivated crop in paddy fields in Korea. Oftentimes, *P. canaliculata* is introduced into the paddy field for removing weeds. *T. tubifex* is a species inhabiting most wetland, such as paddy fields. A mesocosm system consisting of the species can simulate the general paddy ecosystem in Korea.

1.2. Partial Least Squares Path Modeling (PLS-PM)

PLS-PM is a method of structural equation modeling which allows estimating complex cause–effect relationship models with latent variables. The PLS structural equation model comprises two sub-models: the outer model (or measurement model) and inner model (or structural model). The outer model forms latent variable using the related measured variables, whereas the inner model shows the unidirectional paths between the latent variables. In the outer model, the latent variables are expressed as a weighted sum of its manifest (i.e., measured) variables. The measured variables constituting one latent variable should have uni-dimensionality. Dillon–Goldstein's Rho, based on the variance in the sum of measured variables, is used as an indicator for the uni-dimensionality of the measured variables [14]. A threshold value of Dillon–Goldstein's Rho was >0.7 in this study. The composite reliability of the outer model equations is assessed by average variance extracted (AVE) of each latent variable [14]. A threshold value of AVE was >0.5 in this study. In the inner model, causative relationships between the latent variables are described by means of simple or multiple linear regression. In the inner model, the direct cause–effect relationship between the latent variables was obtained by the path coefficients. A path coefficient close to +1 indicates a strong positive causal linkage between variables (and vice versa for negative values). The indirect effect, the causative relationship mediated by intervening variables, was obtained by the product of intervening path coefficients. Thus, the total effect was expressed as the sum of direct and indirect effects [15]. The performance of the overall model can be assessed by the goodness of fit index, the product of validations of the outer model and inner model [16].

In this study, we adopted the structural equation model consisting six latent variables as follows (Table S3): pH_i (initial paddy soil pH measured at the 0th, 1st, and 2nd week), SN4 (soil inorganic nitrogen level at the 4th week), PG4 (plant growth at the 4th week), PG20 (plant growth upon harvest), GY (grain yield), and GQ (grain quality). Between latent variables, the unidirectional path was assumed as follows: the change in pH_i primarily results from the addition of HNO_3 and H_2SO_4. Two latent variables, SN4 and PG4, are affected directly by the change in pH_i. The other three latent variables (i.e., PG20, GY, and GQ) which are related with plant growth and productivity are affected by pH_i, SN4, and PG4. Refer to Table S3 for a list of latent variables and their associated measured variables.

Table S1. Acute toxicities (EC_{50}, mg kg^{-1}) of HNO_3 and H_2SO_4 to five terrestrial species (*Oryza sativa*, *Raphanus sativus*, *Pinus densiflora*, *Paronychiurus kimi*, and *Eisenia fetida*).

Test chemical	Test Species	Test Duration (days)	Endpoint	Toxicity Value [1]
Nitric acid (HNO_3)	*Oryza sativa*	28	Fresh weight of root	1246.71 (729.53–1763.88)
	Raphanus sativus	28	Fresh weight of root	1140.56 (808.79–1472.32)
	Pinus densiflora	32	Leaf area	1378.00 (1242.00–1514.00)
	Paronychiurus kimi	28	Reproduction	578.71 (457.72–699.70)
	Eisenia fetida	2	Growth inhibition	Data not available
Sulfuric acid (H_2SO_4)	*Oryza sativa*	28	Fresh weight of root	Data not available
	Raphanus sativus	28	Fresh weight of root	2123.83
	Pinus densiflora	32	Leaf area	1024.00 (849.00–1198.00)
	Paronychiurus kimi	28	Reproduction	2905.29 (882.42–4928.16)
	Eisenia fetida	2	Growth inhibition	Data not available

[1] Toxicity values are predicted by a non-linear regression model using SAS 9.4 (SAS Institute Inc., USA). Values in the parenthesis are 95% confidence interval.

Table S2. List of toxic endpoints for test species measured at the 4th and 20th week.

**Test species**	**Measured Endpoints**	
	4th week	20th week
Rice (*Oryza sativa*)	Number of surviving hills<br>Chlorophyll content<br>Fresh weight of shoot<br>Plant height<br>Percentage of leaves with eating-damage by *P. canaliculata*<br>Percentage of leaves with wilting damage by acids	Chlorophyll content<br>Fresh weight of shoot<br>Plant height<br>Panicle number<br>Grains number<br>Whole grains weight<br>1000-grain weight<br>Percentage of filled grains
Golden apple snail (*Pomacea canaliculata*)	Survival rate<br>Shell length<br>Weight	-
Sludge worm (*Tubifex tubifex*)	Recovered number of adults<br>Recovered number of juveniles	-

Table S3. List of six latent variables and their corresponding measured variables used in this model. Latent variables are defined by the weighted sum of measured variables (Eqs. S1–S6) [1] as given below.

**Latent variable**	**Measured variable**	**Unit**	**Description**
Initial soil pH (pH_i)	pH-0, pH-1, pH-2	Unitless	Initial paddy soil pH measured at 0, 1, and 2 weeks
Soil nitrogen at the 4th week (SN4)	NH_4^+	mmol kg^{-1}	Concentration of NH_4^+ in paddy soil
	NO_3^-		Concentration of NO_3^- in paddy soil
Plant growth at the 4th week (PG4)	Shoot height (SH-4)	cm	Shoot height of 4-week-old *Oryza sativa*
	Fresh weight (FW-4)	g	Fresh weight of 4-week-old *O. sativa*
Plant growth at the 20th week (PG20)	Shoot height (SH-20)	cm	Shoot height of 20-week-old *O. sativa*
	Fresh weight (FW-20)	g	Fresh weight of 20-week-old *O. sativa*
Grain yield (GY)	Panicle number (PN)	Ea	Number of panicles per hill of 20-week-old *O. sativa*
	Grain number (GN)	Ea	Number of whole grains counted by three hills of 20-week-old *O. sativa*
	Grain weight (GW)	g	Weight of whole grains determined by three hills of 20-week-old *O. sativa*
Grain quality (GQ)	1000-grain weight (1000W)	g	Weight of one thousand grains
	The % of filled grain (FG)	%	Percentage of the number of filled grains to the number of whole grains

[1] Six latent variable are defined as follows. The reliability of measured variables constituting each latent variable was assessed using the Dillon–Goldstein's Rho (D-G Rho) value with a suggested threshold value of 0.7. The validity of the constructed equations was confirmed by the average variance extracted (AVE) of each latent variable, with a recommended threshold value of 0.5 (Sanchez, 2013). Values of D-G Rho and AVE are shown in the parenthesis.

$$pH_i = 0.32\,(pH\text{-}0) + 0.35\,(pH\text{-}1) + 0.36\,(pH\text{-}2) \quad (\text{D-G Rho} = 0.983,\ \text{AVE} = 0.947) \quad \text{Eq. S1} \tag{1}$$

$$SN4 = 0.60\,(NH_4^+) + 0.51\,(NO_3^-) \quad (\text{D-G Rho} = 0.894,\ \text{AVE} = 0.807) \quad \text{Eq. S2} \tag{2}$$

$$PG4 = 0.50\,(SH\text{-}4) + 0.53\,(FW\text{-}4) \quad (\text{D-G Rho} = 0.972,\ \text{AVE} = 0.946) \quad \text{Eq. S3} \tag{3}$$

$$PG20 = 0.43\,(SH\text{-}20) + 0.65\,(FW\text{-}20) \quad (\text{D-G Rho} = 0.923,\ \text{AVE} = 0.853) \quad \text{Eq. S4} \tag{4}$$

$$GY = 0.32\,(PN) + 0.35\,(GN) + 0.35\,(GW) \quad (\text{D-G Rho} = 0.987,\ \text{AVE} = 0.961) \quad \text{Eq. S5} \tag{5}$$

$$GQ = 0.60\,(1000W) + 0.59\,(FG) \quad (\text{D-G Rho} = 0.828,\ \text{AVE} = 0.706) \quad \text{Eq. S6} \tag{6}$$

Table S4. Results [1] of paddy soil analysis at the 4th week.

**Property**	**Control**	**Treatment**			
		HNO_3		H_2SO_4	
		PNEC	HC_{50}	PNEC	HC_{50}
pH	6.47 ± 0.22 a	6.23 ± 0.80 a	3.27 ± 0.27 b	6.65 ± 0.01 a	2.87 ± 0.47 b
SOC	1.72 ± 0.47 a	1.53 ± 0.15 a	1.61 ± 0.05 a	1.42 ± 0.14 a	1.50 ± 0.22 a
NH_4^+	1.43 ± 0.18 a	1.84 ± 0.49 a	2.67 ± 0.39 b	1.62 ± 0.10 a	2.79 ± 0.02 b
NO_2^-	ND	ND	ND	ND	ND
NO_3^-	0.28 ± 0.01 a	0.36 ± 0.07 a	23.4 ± 9.40 b	0.29 ± 0.01 a	0.34 ± 0.07 a
Av-P	0.45 ± 0.08 a	0.49 ± 0.05 a	0.45 ± 0.07 a	0.41 ± 0.11 a	0.37 ± 0.10 a
Av-Si	1.76 ± 0.29 a	1.58 ± 0.11 ab	1.35 ± 0.04 b	1.91 ± 0.04 a	1.33 ± 0.24 b
K^+	3.15 ± 0.31 ab	3.29 ± 0.35 a	2.52 ± 0.17 bc	3.24 ± 0.34 a	2.34 ± 0.53 c

[1] Mean value ± standard deviation. The different letters indicate significant differences among groups at $p \leq 0.05$. Data of the control soil without addition of acid are also shown for comparison. SOC = soil organic carbon (%), Av-P = available phosphorus determined using the Bray No. 1 method. Av-Si = available silicate extracted using 1 M NaOAc solution. ND = not detected. The unit of chemical concentration is mmol kg^{-1}.

Table S5. Pearson correlation coefficient [1] (r) between rice productivity and chlorophyll content [2] as a function of growth stage.

Growth stage		Grain Yield			Grain Quality	
		Panicle number	Grain number	Whole grain weight	1000-grain weight	Filled grain (%)
Vegetative phase (0th–8th week)	Active tillering stage	0.28	0.28	0.26	− 0.18	0.28
	Non-productive tillering stage	0.64*	0.78**	0.79***	− 0.61*	–0.59*
Reproductive phase (9th–12th week)	Booting, Heading, and Flowering stage	0.66*	0.73**	0.75**	–0.55*	–0.77***
Ripening phase (13th–20th week)	Grain filling stage	0.67**	0.64*	0.67**	–0.47	–0.90***
	Maturity stage	0.69**	0.62*	0.66**	–0.45	–0.89***

[1] The number of asterisks indicates the significance level of correlation: * $p \leq 0.05$, ** $p \leq 0.01$, and *** $p \leq 0.001$. Correlations without an asterisk are not statistically significant ($p > 0.05$).

[2] Average values of chlorophyll content in leaf measured at the given growth stage.

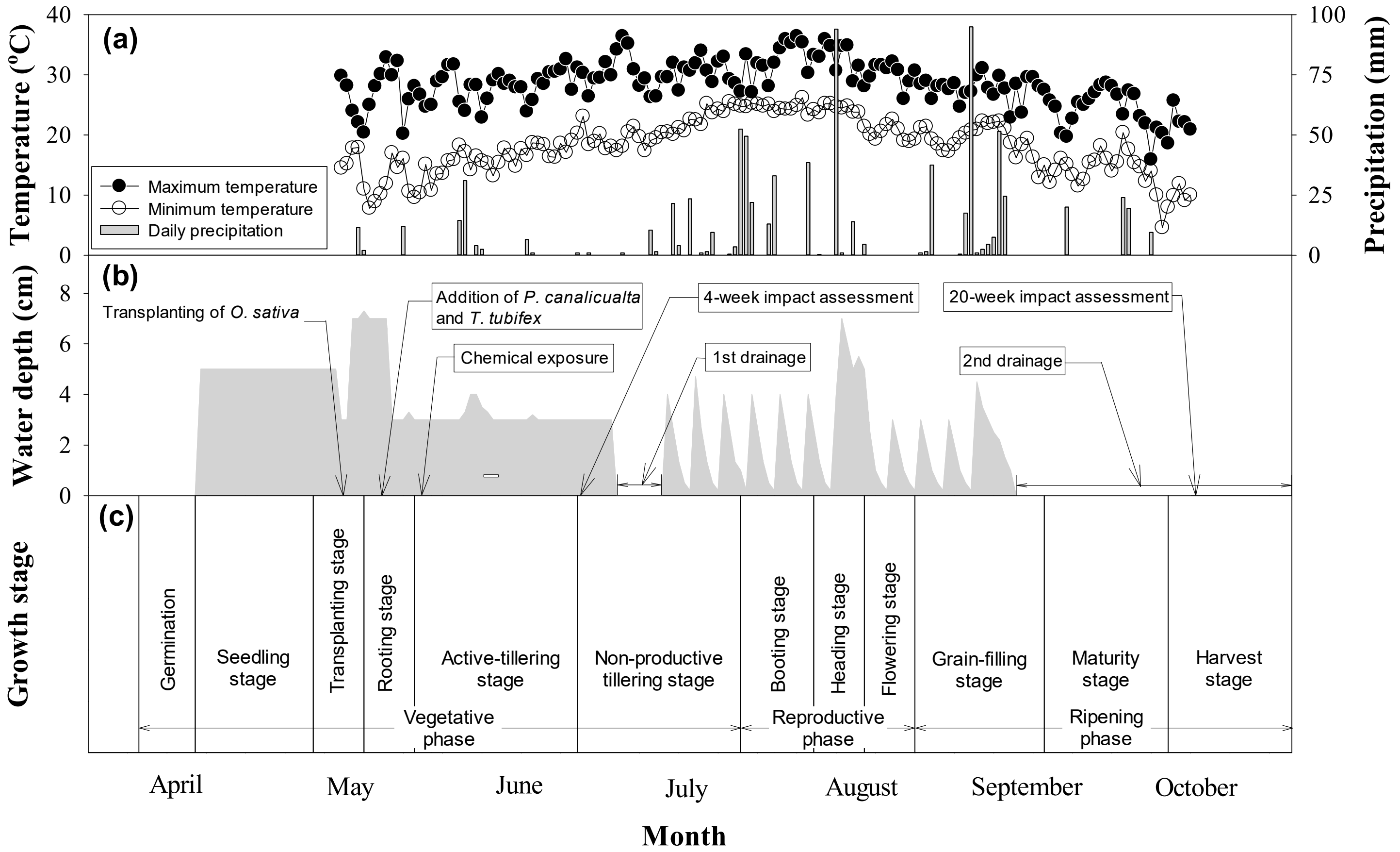

Figure S1. Timewise condition during the 20-week experimental period: (a) precipitation and temperature, (b) the level of paddy water along with the timing of test species and test chemicals introduction, and (c) life stage of rice (*Oryza sativa*) cultivation during the cropping season (from germination to harvest).

© 2020 by the authors. Licensee MDPI, Basel, Switzerland. This article is an open access article distributed under the terms and conditions of the Creative Commons Attribution (CC BY) license (http://creativecommons.org/licenses/by/4.0/).
